# *GATA2* Is Associated with Familial Early-Onset Coronary Artery Disease

**DOI:** 10.1371/journal.pgen.0020139

**Published:** 2006-08-25

**Authors:** Jessica J Connelly, Tianyuan Wang, Julie E Cox, Carol Haynes, Liyong Wang, Svati H Shah, David R Crosslin, A. Brent Hale, Sarah Nelson, David C Crossman, Christopher B Granger, Jonathan L Haines, Christopher J. H Jones, Jeffery M Vance, Pascal J Goldschmidt-Clermont, William E Kraus, Elizabeth R Hauser, Simon G Gregory

**Affiliations:** 1 Department of Medicine and Center for Human Genetics, Duke University Medical Center, Durham, North Carolina, United States; 2 Department of Medicine and Division of Cardiology, Duke University Medical Center, Durham, North Carolina, United States; 3 Cardiovascular Research Group, Northern General Hospital, University of Sheffield, Sheffield, United Kingdom; 4 Center for Human Genetics Research and Department of Molecular Physiology and Biophysics, Vanderbilt University, Nashville, Tennessee, United States; 5 University of Wales College of Medicine, Cardiff, United Kingdom; 6 Miller School of Medicine, University of Miami, Miami, Florida, United States; University of Oxford, United Kingdom

## Abstract

The transcription factor GATA2 plays an essential role in the establishment and maintenance of adult hematopoiesis. It is expressed in hematopoietic stem cells, as well as the cells that make up the aortic vasculature, namely aortic endothelial cells and smooth muscle cells. We have shown that *GATA2* expression is predictive of location within the thoracic aorta; location is suggested to be a surrogate for disease susceptibility. The *GATA2* gene maps beneath the Chromosome 3q linkage peak from our family-based sample set (GENECARD) study of early-onset coronary artery disease. Given these observations, we investigated the relationship of several known and novel polymorphisms within *GATA2* to coronary artery disease. We identified five single nucleotide polymorphisms that were significantly associated with early-onset coronary artery disease in GENECARD. These results were validated by identifying significant association of two of these single nucleotide polymorphisms in an independent case-control sample set that was phenotypically similar to the GENECARD families. These observations identify *GATA2* as a novel susceptibility gene for coronary artery disease and suggest that the study of this transcription factor and its downstream targets may uncover a regulatory network important for coronary artery disease inheritance.

## Synopsis

Coronary artery disease (CAD) is the most common form of heart disease in the Western world and is one of the leading causes of death in the United States. CAD is inherited and is a complex genetic disease because it results from changes to multiple genes acting in concert with one another and the environment. The authors locate CAD susceptibility genes by convergence of techniques and identify variation within a gene of interest in an early-onset CAD population. If a specific variant is found more often in affected individuals or families than in controls, this can suggest that this gene variant is associated with disease. The authors have identified a gene, *GATA2,* which is located in a genomic region suspected to contain genes for CAD and displays expression patterns predictive of location of disease within human donor aortas. They have identified several *GATA2* variants that segregate with CAD in a family-based early-onset CAD population and have further validated two of these associations in a separate young case-control sample affected with CAD. These data imply that the transcription factor GATA2 may play a role in CAD susceptibility and suggest that the study of GATA2 targets may uncover a set of GATA2-regulated genes important to CAD inheritance.

## Introduction

Coronary artery disease (CAD) is the most common form of heart disease in the Western world. It affects more than 13 million Americans and is one of the leading causes of death in the United States [1]. CAD is a complex genetic disease; despite substantial evidence of a genetic contribution for CAD and its risk factors [2–10], the mode of inheritance does not follow Mendelian segregation. Complex genetic diseases are considered multifactorial in that they are characterized by the inheritance of multiple genetic variants acting in concert with environmental effects to promote the disease state.

Despite the obvious importance of environmental and behavioral risk factors, evidence of the genetic contribution to CAD is strong and consistent. The estimated relative risk of developing early-onset CAD in a first-degree relative is between 3.8 and 12.1, depending on the age of onset (AOO) of the proband, and higher risk correlates with earlier AOO [2,10]. Additionally, data from the Framingham Heart Study have shown an increased risk of incident cardiovascular disease in both age-sex–adjusted models (odds ratio [OR] = 1.55) and models adjusted for cardiovascular risk factors (OR = 1.45) for siblings of affected individuals [4].

Ten separate linkage screens have been performed in CAD sample sets to identify candidate genes that contribute to the genetic etiology of CAD, including our own GENECARD study [11]. Cumulatively, these screens have identified regions of linkage on Chromosomes 1, 2, 3, 4, 5, 7, 12, 13, 14, 16, 17, 19, and X [11–20], with genetic regions on 3q26–27 and 2q34–37 displaying cross-study CAD susceptibility [21]. Additionally, a recent genome screen by Bowden et al. [20] identified linkage to Chromosome 3q13 in a type 2 diabetes population subset by self-reported and clinically assessed CAD (The Diabetes Heart Study). This region is nearly identical to the Chromosome 3q13 linkage identified in the GENECARD study. Though a collection of CAD genetic regions have been identified, only a single gene mapping to Chromosome 13q, *ALOX5AP,* has been shown to contain variants that reproducibly predispose individuals to myocardial infarction, a specific manifestation of the cardiovascular disease phenotype [15,22–24].

Our goal is to characterize the underlying genetic mechanisms involved in the development of CAD. Through convergent analyses of linkage and expression data, we have identified the transcription factor *GATA2* as having an increased potential to be involved with CAD susceptibility. We utilized linkage data from our GENECARD genome-wide linkage study that sampled a cohort of families with at least two siblings with early-onset CAD (AOO in men <51 y and women <56 y) [25]. The most significant region of linkage, of the several regions that were identified [11], was localized to Chromosome 3q13 (multipoint log of the odds [LOD] = 3.50). *GATA2* maps beneath the one-LOD down support interval for this linkage peak on Chromosome 3.

We also utilized gene expression patterns from Seo et al. [26] that identified *GATA2* as one of the genes most predictive of the location within human donor aortas. Given that atherosclerotic disease has been shown to display increasing severity from proximal to distal locations within the aorta [27], these investigators theorized that differences in regional expression patterns within the aorta could be related to disease susceptibility.

Given the convergence of the results, we hypothesized that *GATA2* may represent a newly identified susceptibility gene for CAD. Therefore, we investigated the haplotype structure of *GATA2* to identify single nucleotide polymorphisms (SNPs) for genotyping within the gene in order to test the hypothesis that *GATA2* is associated with CAD. We identified association in both a family-based sample (GENECARD) and in a validation dataset of nonfamilial CAD (CATHGEN). The results identify *GATA2* as a novel CAD susceptibility gene and suggest that the study of this transcription factor and its downstream targets may undercover a regulatory network important for CAD.

## Results

### *GATA2* SNP Selection and Genotyping

**Figure 1:**
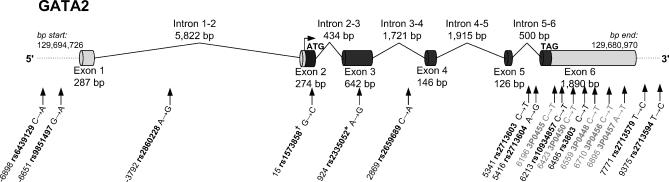
Schematic of the *GATA2* Gene Structure The 12 SNPs representing predicted LD bins in *GATA2* are shown in black; the five novel SNPs identified through sequencing are shown in grey. † and * indicate a synonymous and nonsynonymous SNP, respectively.

HapMap (http://www.hapmap.org) and Perlegen (http://genome.perlegen.com) databases were used to identify known SNPs within the *GATA2* gene and 3,000 base pairs both upstream and downstream of the gene to account for putative promoter and downstream regulatory elements. A total of 12 SNPs were identified with a minor allele frequency (MAF) >10% in the Caucasian population (white Americans of European descent). LDSelect [28], which is used by SNPselector [29], determined that these 12 SNPs, using an r^2^ = 0.7, represent 12 linkage disequilibrium (LD) bins. All SNPs, with the exception of rs1806462, were genotyped in our GENECARD sample set (Figure 1, LD bin SNPs are black). Concurrently, we sought to determine whether additional coding SNPs within *GATA2* could be identified. We sequenced each of the six *GATA2* exons using a Caucasian sample consisting of 16 affected individuals and 16 individuals of unknown status. Five novel SNPs were identified within the 3′ untranslated region of the gene, whereas three previously validated SNPs were identified and two additional SNPs, which did not have a validated status in dbSNP (rs11708606 and rs10934857), were validated by our novel sequence (Table 1). We included rs10934857 in our analysis because LD calculations using the sequenced individuals suggested that this SNP may reside in a separate bin. The location of the five novel SNPs in relation to the 12 selected SNPs for our association studies is shown in Figure 1 (novel SNPs are grey). All 17 SNPs were genotyped in our association studies.

**Table 1:**
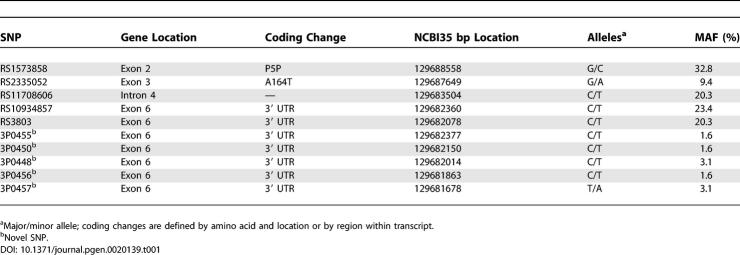
SNPs Identified by Sequencing the Six Exons of *GATA2* in 32 Individuals Including Five Novel *GATA2* SNPs

### Single-Marker Family-Based Association in GENECARD

**Figure 2:**
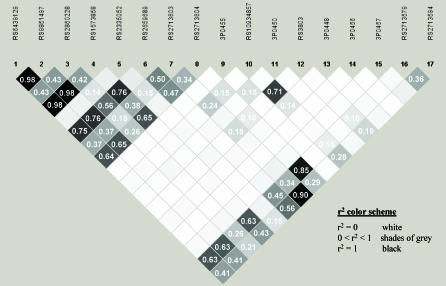
Pairwise LD between *GATA2* SNPs LD was estimated in one unaffected Caucasian individual from each nonredundant GENECARD discordant sibling pair (*n* = 279). A similar pattern of LD was observed using the matched probands.

Genotyping was performed on the 12 a priori SNPs and the five novel SNPs in the GENECARD sample, which represents both the original genome linkage screen families as well as the follow-up collection set (Table 2, *n* = 1,101 families). The characteristics of this study group are defined in Tables 2 and 3 and elsewhere [11,25]. Pairwise LD between each SNP was measured using the Graphical Overview of Linkage Disequilibrium package separately in both Caucasian affected (GENECARD probands with unaffected siblings) and unaffected (proband matched unaffected) individuals; no significant differences were seen between these two groups (unpublished data). We examined the LD structure of *GATA2* in order to assess the quality of our tagSNP selection. The Haploview plot of these data in the unaffected Caucasian population (Figure 2) shows a weak block of LD in the 5′ end of the gene. The lack of haplotype block structure from our analysis was expected because of the prior selection of nonredundant haplotype tagging SNPs.

**Table 2:**
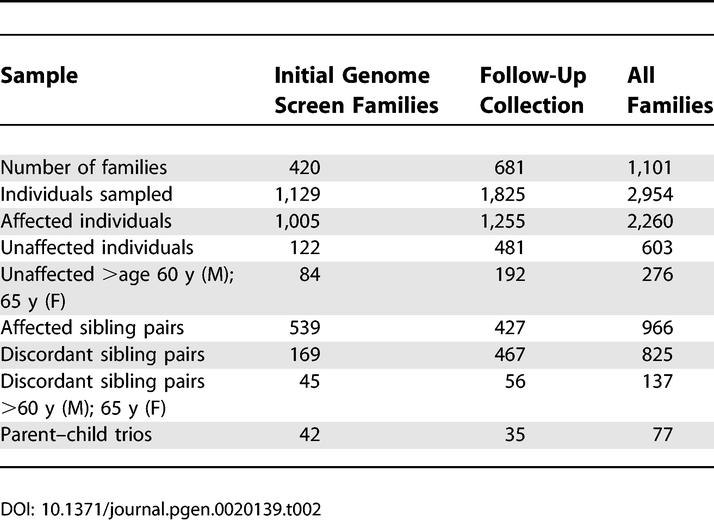
GENECARD Sample Size

**Table 3:**
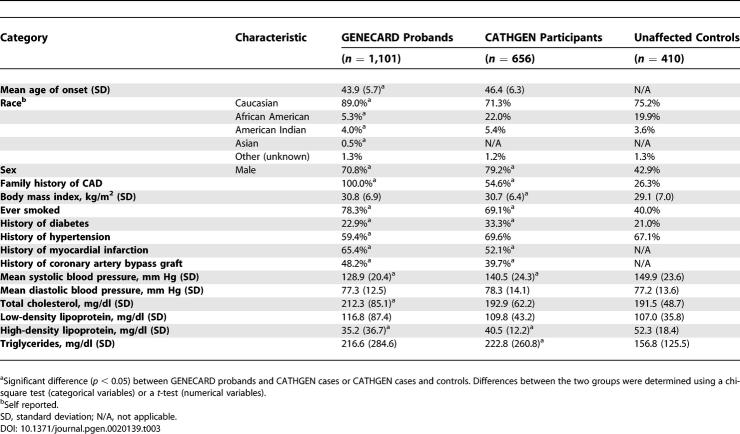
Clinical Characteristics of GENECARD Probands and CATHGEN Participants

We employed the test for association in the presence of linkage (APL) to conduct association analysis at markers in *GATA2* in order to make use of the large number of affected sibling pairs in the GENECARD sample, as well as to appropriately infer missing parental genotypes and to account for the correlation between transmission of parental marker alleles to multiple affected offspring due to linkage [30]. APL analysis of the 17 *GATA2* SNPs identified five significant associations (*p* < 0.05) with early-onset CAD (Table 4, highlighted in red). Four of the five associated SNPs are located in the distal end of *GATA2*, encompassing intron 5, exon 6, and a region downstream of the gene. Two of these SNPs are in moderate LD with one another, rs2713604 and rs2713579 (r^2^ = 0.85). We noted that none of these SNPs would withstand the stringent Bonferroni correction for multiple comparisons (α = 0.05, *n* = 17, *p* ≤ 0.003). Additionally, we assessed evidence for linkage to the Chromosome 3 region in the additional GENECARD families from the follow-up collection. The multipoint LOD scores for the *GATA2* region including the flanking microsatellites are zero. The largest two-point result in the second GENECARD population is 0.39 for rs2335052. Thus, there appears to be no strong and consistent evidence for linkage as we observed in the initial genome screen families.

**Table 4:**
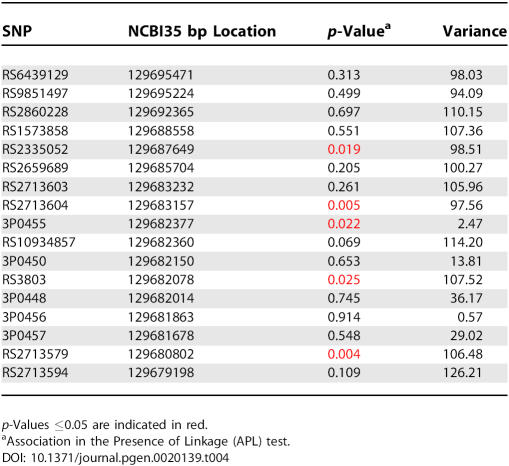
*GATA2* SNPs Are Associated with Early-Onset CAD in GENECARD

### Haplotype Analysis of *GATA2* SNPs in GENECARD

We have identified a region of association with CAD within the 3′ end of *GATA2*, which encompasses four SNPs. In order to more accurately identify the SNP(s) associated with early-onset CAD, we used the APL test to calculate the transmission frequencies of all possible haplotypes for pairwise combinations of the 17 SNP markers within *GATA2* in the GENECARD sample. These frequencies were compared in a global test accounting for rare haplotypes [30] (Table S1). We observed six pairwise haplotypes of the 171 comparisons made, which involved seven SNPs with little to no LD (0.02 < r^2^ < 0.28) with remarkably strong associations (Table 5). These six pairs maintained strong association after Bonferroni correction, with rs2713604_rs3803 and rs3803_rs2713594 exhibiting the strongest association (global Bonferroni corrected *p* < 0.0012 for both).

**Table 5:**
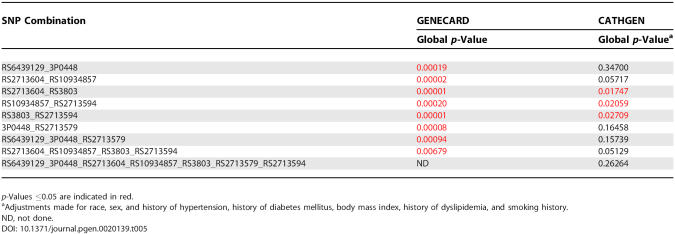
Haplotype Analysis Identifies Significant Haplotypes Associated with Early-Onset CAD

The pairwise haplotype results suggest that these haplotypes may be extended to include additional SNPs. We identified a single three-SNP haplotype and an independent four-SNP haplotype using the strongly associated haplotype pairs that shared a common SNP. These haplotypes were examined using the APL test. Both of these haplotypes were overtransmitted in GENECARD families (Table 5). We were not able to analyze the full seven SNP haplotypes in family-based APL analysis.

### Replicating Single-Marker Association of *GATA2* SNPs in CATHGEN Case-Control Sample

We identified five SNPs within *GATA2*, as well as six pairwise haplotypes and a single three-SNP and four-SNP haplotype that are significantly associated with early-onset CAD. We identified 656 cases and 410 controls meeting the study criteria (see Materials and Methods) from the CATHGEN study to validate these results in a phenotypically similar, nonfamilial young affected CAD case-control cohort. Baseline clinical characteristics in GENECARD probands (*n* = 1,101) and CATHGEN participants and unaffected controls are presented in Table 3. We genotyped the five SNPs significantly associated with disease status and the additional four SNPs that were identified in the haplotype analysis, in the CATHGEN cases and controls. Allelic association was examined using a multivariable logistic regression analysis. In order to test for a true genetic effect, we adjusted for race and sex or race, sex, and known CAD risk factors (see Materials and Methods). We identified significant associations in two of the five SNPs, rs2713604 and rs3803 (Table 6), with stronger association identified in the race, sex, and CAD risk factor–adjusted model (compare Table 6 and Table S2). Moreover, we identified the minor allele of rs2713604 as a risk allele (OR = 1.52, 95% confidence interval [CI] = 1.10 to 2.09) and the minor allele of rs3803 as a protective allele (OR = 0.69, 95% CI = 0.50 to 0.96). In order to confirm the direction of the association of these two alleles in GENECARD, we compared the GENECARD probands from the United States to the CATHGEN controls. We verified that the allele frequencies are similar to those observed for the CATHGEN cases (Table S3); in addition, we observed similar odds ratios, though rs2713604 is not significant.

**Table 6:**
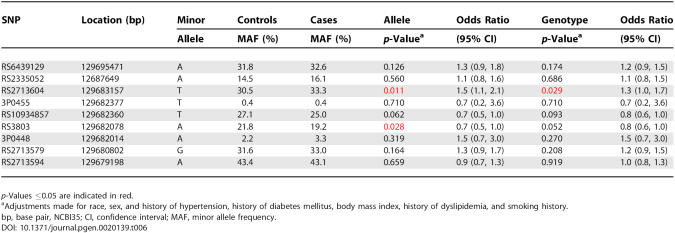
Replication of *GATA2* SNPs Associated with Early-Onset CAD in the CATHGEN Case-Control Sample Set

### Haplotype Analysis in CATHGEN

The six significant pairwise haplotypes from the GENECARD analysis suggest that these haplotypes may be important in defining CAD risk; we performed haplotype analysis in the CATHGEN population. We examined the six significant pairwise haplotypes from GENECARD in the CATHGEN population and identified three significant (*p* < 0.05) pairs (Table 5). We expanded our analysis to include the three-SNP haplotype, the four-SNP haplotype, and the complete seven-SNP haplotype suggested by the GENECARD sample, with global *p*-values of 0.16, 0.05, and 0.26, respectively (Table 5).

We were intrigued by the most significant haplotype in GENECARD and CATHGEN, rs2713604_rs3803, because the results from the logistic regression suggested that the minor allele of rs2713604 conferred risk, whereas the minor allele of rs3803 was protective. Upon further analysis, we discovered that the rs2713604_T_rs3803_A haplotype (risk_protective), which is predicted to occur at 6.5% in the population, does not exist in either of our populations. Although the LD as measured by r^2^ is low, the D′ value is nearly 1, reflecting the complete absence of the haplotype involving the associated alleles at markers rs2713604 and rs3803 (Table S4). The two-locus genotypes confirm that this haplotype is not observed in our data. Additionally, the absence of these genotype combinations makes the statistical analysis of possible multilocus or interaction effects nearly impossible.

## Discussion

We identified a transcription factor, *GATA2*, through convergent analysis of linkage and expression data in an effort to define the underlying molecular mechanisms that lead to CAD. Genotyping and subsequent analysis of *GATA2* tagging and novel SNPs in a family-based early-onset CAD sample identified five SNPs significantly associated with early-onset CAD. We validated the association of two of these SNPs, rs2713604 and rs3803, in an independent case-control dataset, as well as the direction of the association, thus identifying *GATA2* as a susceptibility gene candidate for early-onset CAD.

The data suggest that polymorphisms in the 3′ end of *GATA2* may increase susceptibility to developing CAD. We identified several novel SNPs within the sixth exon of *GATA2* but did not identify association with early-onset CAD or LD between these SNPs and SNPs that were found to be associated with early-onset CAD. Although the functional relevance of our two most significantly associated SNPs remains unknown, one of these SNPs, rs3803, is conserved between multiple species (human, mouse, rat, and dog) and lies near one of three known polyadenylation sites [31,32]. We are currently investigating whether any of these polymorphisms control tissue-specific polyadenylation of this transcript.

We searched for haplotypes associated with disease using the GENECARD and CATHGEN cohorts. We identified several pairwise haplotypes in GENECARD, three of which were significant in CATHGEN; however, we were unable to identify a single consistent haplotype combination when we expanded these haplotypes to include four SNPs. There are at least three possible explanations for these results. The analysis in the CATHGEN group may be underpowered when additional markers are added to the haplotypes, thus creating additional rare haplotypes. There may also be an ungenotyped SNP in weak LD with both haplotype sets, which would explain the finding of association with two separate sets of SNPs. A third explanation for these findings is that there is no single haplotype underlying the increased risk for CAD, but rather there are multiple SNPs and potentially multiple independent haplotypes. A larger sample size and additional genotyping will be required to evaluate these scenarios. Although the haplotype results do not point to a consistent risk haplotype within the *GATA2* gene, taken together our results identify nucleotide variants within *GATA2* as a risk factor of early-onset CAD.

One of the more difficult aspects of genetic association analysis is the appropriate multiple comparison correction of the statistical significance of any given result. These corrections range from the most conservative Bonferroni correction, to false discovery rate approaches [33], to weighted corrections of combined data, to no correction at all. In our case we report uncorrected *p*-values; however, it should be noted that the results for the haplotype analysis would, in fact, survive a Bonferroni correction. We base our enthusiasm for *GATA2* as a gene implicated in CAD susceptibility because we identified two separate significant markers in low LD in two independent populations. We believe that it is highly likely that they constitute two important independent markers for two reasons: they are physically linked within the same gene and in the same part of the gene and are significantly associated with disease in two independent populations. Further work to elucidate the function of GATA2 in CAD, including the CAD-associated polymorphisms and its downstream targets, is necessary in order to begin to understand the role this transcription factor plays in this disease.

Genetic and phenotypic heterogeneity are features of complex disease, particularly CAD. The pathobiology of CAD itself is sufficiently intricate in that the core of the disease is the formation of the atherosclerotic plaque. The pathways that are involved in lesion predisposition, formation, and disruption are numerous and can be modulated by the environment and other underlying genetic diseases (for example, diabetes or lipid disorders), as reviewed by Watkins and Farrall [34]. Additionally, the lack of a consistent clinical definition of CAD across studies further confounds genetic analysis. The detection of multiple, significant but non-overlapping chromosomal regions in ten genome screens is an indicator of the mixed genetic and phenotypic characteristics of this disease. We are encouraged by the recent data of Bowden et al. [20] that suggest a replication of the Chromosome 3q13 linkage evidence we initially reported, though phenotypic heterogeneity still remains. We also see evidence of this heterogeneity in our own dataset, both phenotypic [35] and in the linkage scores. We failed to detect evidence for linkage on Chromosome 3 in our follow-up dataset (maximum multipoint LOD score = 0.0 at *GATA2*), and though the result may be discouraging, the result is not surprising given the observed heterogeneity of linkage evidence across the ten previously published genome screens. Despite the evident heterogeneity in our CAD population, we detected an association signal with SNPs in *GATA2* in two separate CAD datasets. We suggest that our validation of *GATA2* warrants further study in our datasets, including an expansion to other racial groups such as Asians and African Americans, as well as in other studies to understand what role GATA2 might play in CAD development.

The results of our study are important for three main reasons. First, GATA2 is a transcription factor that is indispensable for all hematopoiesis [36]. It is essential for the development and differentiation of hematopoietic stem and progenitor cells [36–38]. It is expressed both in endothelial cells [37] and smooth muscle cells (J. Connelly, unpublished data), the two primary cell types comprising the aorta. It has been shown to regulate *EDN1* transcription, a potent vasoconstrictor that is expressed only in endothelial cells [39]. It is also known to regulate several other endothelial-specific genes, namely *NOS3* [40], *VWF* [41], *KDR* [42], and *PECAM1* [43], each of which can be linked to CAD [44–50]. The role GATA2 plays in hematopoiesis and endothelial cell function suggests that GATA2 may participate in endothelial progenitor cell potential and, thus, vascular disease propensity [51–55]. The effect of the CAD-associated polymorphisms on GATA2 function still remains to be elucidated. However, our data suggest that GATA2 may be functionally involved in the pathophysiology of CAD.

Second, our study results are important because of the strength of identifying significant associations with complex disease arising from a hypothesis-driven experimental design. We identified and genotyped tagging SNPs in a candidate gene that was identified through “genomic convergence” [56], a convergence of multiple lines of evidence. We used a family-based sample set that is heavily laden with a genetic burden for CAD, as well as a test of association (APL) that allows us to make full use of the GENECARD population while accounting for linkage. We selected a phenotypically similar case-control dataset to validate the *GATA2* association we observed in GENECARD, which allowed us to identify two strong and separate associations with CAD. The use of convergence for candidate gene selection coupled with the thorough coverage of LD across a gene has allowed us to identify two significantly associated SNPs in two independent early-onset CAD samples.

Third, two other transcription factors have been implicated in cardiovascular phenotypes, *MEF2A* (myocardial infarction) [57–59] and *USF1* (lipid traits) [60–62]. Complex genetic diseases, such as cardiovascular disease, are most likely the result of an accumulation of multiple small genetic changes that influence an individual's ability to cope with biological and environmental effects. Transcription factors (and their cognate binding sites), which ultimately influence the expression of many downstream genes, may be important targets to characterize when considering how small genetic changes can influence multiple genetic outcomes. Slight changes to the level of a transcription factor in the cell can have a dramatic effect on the downstream targets of these factors. Hence, these types of genes will most likely be important in the dissection of complex human disease.

Our work suggests that common variants within *GATA2* play a role in CAD, an important complex genetic disease. Identification of a transcription factor associated with CAD in two separate samples implies that GATA2-regulated genes may play a very significant role in CAD susceptibility and progression. These GATA2 target genes, which remain to be explored in this context, represent a rich set of candidate genes from which to dissect genetic contributions to coronary disease susceptibility.

## Materials and Methods

### Early-onset CAD family-based sample (GENECARD).

GENECARD is a collaborative study involving investigators affiliated with the Duke Center for Human Genetics, the Duke University Center for Living, the Duke Clinical Research Institute, the Duke University Consortium for Cardiovascular Studies, and additional investigative sites of the GENECARD Study Network. The study is coordinated at Duke and located throughout five other international sites, and the study design has been previously reported [25]. In brief, collection of families began in March 1998 and was completed on 31 March, 2002. All study participants signed a consent form approved by the responsible institutional review board or local ethics committee.

The sample set used for the initial genome-wide linkage screen within the GENECARD project was composed of 493 affected sibling pairs in 420 families, where at least two siblings met the criteria for early-onset CAD. The characteristics of this study group are summarized in Table 2 and elsewhere [11,25]. We have expanded our collection to include an additional 681 families together with a large number of unaffected participants from all families. Unaffected participants were defined as siblings and relatives who have not been diagnosed with CAD and are older than 55 y of age (males) or older than 60 y of age (females). This additional collection has increased our sample size to 2,954 affected and unaffected individuals (Table 2).

### Early-onset CAD case-control sample (CATHGEN).

CATHGEN participants were recruited sequentially through the cardiac catheterization laboratories at Duke University Hospital (Durham, North Carolina, United States) with approval from the Duke Institutional Review Board. All participants undergoing catheterization were offered participation in the study and signed informed consent. Medical history and clinical data were collected and stored in the Duke Information System for Cardiovascular Care database maintained at the Duke Clinical Research Institute [63].

Controls and cases were chosen on the basis of extent of CAD as measured by the CAD index (CADi). CADi is a numerical summary of coronary angiographic data that incorporate the extent and anatomical distribution of coronary disease [64]. CADi has been shown to be a better predictor of clinical outcome than extent of CAD [65]. Affected status was determined by the presence of significant CAD defined as a CADi ≥ 32 [66]. For patients older than 55 y of age, a higher CADi threshold (CADi ≥ 74) was used to adjust for the higher baseline extent of CAD in this group. Medical records were reviewed to determine the AOO of CAD, i.e., the age at first documented surgical or percutaneous coronary revascularization procedure, myocardial infarction, or cardiac catheterization meeting the above-defined CADi thresholds. The CATHGEN cases were stratified into a young affected group (AOO ≤ 55 y), which provides a consistent comparison for the GENECARD family study. Controls were defined as ≥60 y of age, with no CAD as demonstrated by coronary angiography and no documented history of cerebrovascular or peripheral vascular disease, myocardial infarction, or interventional coronary revascularization procedures. A comparison of clinical characteristics between GENECARD and CATHGEN probands and unaffected CATHGEN controls is presented in Table 3.

### Novel SNP detection by *GATA2* re-sequencing.

*GATA2* exons (six in total) were PCR amplified using standard conditions and sequenced using ABI Big Dye v3.1 and an ABI 3730 automated sequencer. Sequencing data were analyzed using Sequencher software (Gene Codes, Ann Arbor, Michigan, United States). All sequence amplicons were generated within 16 GENECARD affected individuals and 16 randomly ascertained individuals of unknown status. DNA derived from the blood of 17 Caucasian males and 15 Caucasian females was used for novel SNP discovery.

### SNP genotyping.

A minimal set of tagging SNPs with an MAF of >10% [28] was selected for genotyping in the GENECARD and CATHGEN samples to cover the predicted LD structure in *GATA2* (~14 kilobases) using the SNPselector program [29]. Additionally, five novel *GATA2* SNPs identified by de novo sequencing were also genotyped in the GENECARD and CATHGEN collections. Genomic DNA for the GENECARD and CATHGEN samples was extracted from whole blood using the PureGene system (Gentra Systems, Minneapolis, Minnesota, United States). Genotyping in GENECARD was performed using the ABI 7900HT Taqman SNP genotyping system (Applied Biosystems, Foster City, California, United States), which incorporates a standard PCR-based, dual fluor, allelic discrimination assay in a 384-well–plate format with a dual laser scanner. Allelic discrimination assays were purchased through Applied Biosystems, or, if the assays were not available, primer and probe sets were designed and purchased through Integrated DNA Technologies (Coralville, Iowa, United States). A total of 15 quality control samples—composed of six reference genotype controls in duplicate, two Centre d'Etude du Polymorphisme Humain pedigree individuals, and one no-template sample—were included in each quadrant of the 384-well plate. Genotyping in CATHGEN was performed using the Illumina BeadStation 500G SNP genotyping system (Illumina, San Diego, California, United States). Each Sentrix Array generates 1,536 genotypes for 96 individuals; within each individual array experiment, four quality control samples were included, two Centre d'Etude du Polymorphisme Humain pedigree individuals and two identical in-plate controls. Results of the Centre d'Etude du Polymorphisme Humain and quality-control samples were compared to identify possible sample plating errors and genotype calling inconsistencies. SNPs that showed mismatches on quality-control samples were reviewed by an independent genotyping supervisor for potential genotyping errors. All SNPs examined were successfully genotyped for 95% or more of the individuals in the study. Error rate estimates for SNPs meeting the quality control benchmarks were determined to be less than 0.2%.

### Statistical analysis.

All SNPs were tested for deviations from Hardy-Weinberg equilibrium in the affected and unaffected race-stratified groups. No such deviations were observed. Additionally, LD between pairs of SNPs was assessed using the Graphical Overview of Linkage Disequilibrium package [67] and displayed using Haploview [68]. Family-based association was tested using the APL test [30]. The APL test incorporates data from affected sibling pairs with available parental data and unaffected siblings in the analyses, effectively using all available information in the GENECARD families. The APL software appropriately accounts for the non-independence of affected siblings and calculates a robust estimate of the variance. APL results from markers with variance estimates of less than five are viewed as less reliable [69]. Allelic association in CATHGEN and the GENECARD probands from the United States was examined using multivariable logistic regression modeling adjusted for race and sex, and also for race, sex, and known CAD risk factors (history of hypertension, history of diabetes mellitus, body mass index, history of dyslipidemia, and smoking history) as covariates. These adjustments could hypothetically allow us to control for competing genetic pathways that are independent risk factors for CAD, thereby allowing us to detect a separate CAD genetic effect. SAS 9.1 (SAS Institute, Cary, North Carolina, United States) was used for statistical analysis. APL software was used to identify haplotypes in GENECARD. The HaploStats package was used to identify and test for association of haplotypes in CATHGEN. HaploStats expands on the likelihood approach to account for ambiguity in case-control studies by using a generalized linear model to test for haplotype association, which allows for adjustment of nongenetic covariates [70]. This method derives a score statistic to test the null hypothesis of no association of the trait with the genotype. In addition to the global statistic, HaploStats computes score statistics for the components of the genetic vectors, such as individual haplotypes. The software MERLIN (Multipoint Engine for Rapid Likelihood Inference) was used for two-point and multipoint nonparametric linkage analysis [71].

## Supporting Information

Table S1 Pairwise Haplotype Analysis within GENECARD Identifies Six Significant SNP Pairs in *GATA2*
(19 KB XLS)

Table S2 Replication of *GATA2* SNPs Associated with Early-Onset CAD Adjusting for Race and Sex (15 KB XLS)

Table S3 Replication of Allele Direction of rs2713604 and rs3803 in GENECARD (15 KB XLS)

Table S4 The Risk and Protective Alleles of rs2713604 and rs3803 Do Not Occur on the Same Haplotype (15 KB XLS)

### Accession Numbers

The HUGO Gene Nomenclature Committee (http://www.gene.ucl.ac.uk/nomenclature) accession numbers for the genes and gene products mentioned in this paper are *ALOX5AP* (436), *EDN1* (3176), *GATA2* (4171), *KDR* (6307), *MEF2A* (6993), *NOS3* (7876), *PECAM1* (8823), *USF1* (12593), and *VWF* (12726).

The Online Mendelian Inheritance in Man (http://www.ncbi.nlm.nih.gov/entrez/query.fcgi?db=OMIM) accession numbers for the genes and gene products mentioned in this paper are *ALOX5AP* (603700), *EDN1* (131240), *GATA2* (137295), *KDR* (191306), *MEF2A* (600660), *NOS3* (163729), *PECAM1* (173445), *USF1* (191523), and *VWF* (193400).

## Abbreviations

AOO: age of onset
APL: association in the presence of linkage
CAD: coronary artery disease
CADi: coronary artery disease index
LD: linkage disequilibrium
LOD: log of the odds
MAF: minor allele frequency
OR: odds ratio
SNP: single nucleotide polymorphism
